# Clinical Comparison of Guided Biofilm Therapy and Scaling and Root Planing in the Active Phase of Periodontitis Management

**DOI:** 10.1055/s-0044-1791221

**Published:** 2024-11-07

**Authors:** Magda Mensi, Annamaria Sordillo, Silvia Marchetti, Stefano Calza, Eleonora Scotti

**Affiliations:** 1 Section of Periodontics, School of Dentistry, Department of Surgical Specialties, Radiological Science and Public Health, University of Brescia, Brescia, Italy; 2 U.O.C. Odontostomatologia - ASST degli Spedali Civili di Brescia, Brescia, Italy; 3 Department of Molecular and Translational Medicine, University of Brescia, Brescia, Italy

**Keywords:** periodontitis, periodontal, debridement, dental scaling, root planing

## Abstract

**Objective** The aim of this randomized, controlled, split-mouth study was to compare full-mouth air polishing followed by ultrasonic debridement (known as Guided Biofilm Therapy [GBT]) versus traditional Scaling and Root Planing (SRP), in terms of pocket closure in patients with stages III and IV periodontitis.

**Materials and Methods** The patients underwent periodontal therapy in two sessions. At the beginning of the first session, quadrants I and IV and II and III were randomly assigned to GBT or SRP treatment. Periodontal parameters were collected at baseline, 6 weeks (T1), and 3 months (T2) after therapy. The primary outcome was the number of experimental sites (pocket probing depth [PPD] >4 and <10 mm) becoming closed pockets (PPD ≤ 4 mm bleeding on probing [BOP] negative) at T1 and T2. Secondary outcomes were PPD, recession, clinical attachment level, BOP, and plaque index variations at the experimental sites and treatment time.

**Statistical Analysis** A 10% difference in the primary outcome between the two protocols was set as the threshold to define inferiority/noninferiority of the test treatment. The primary outcome was modeled using a generalized estimating equation model to account for intrapatient measurement correlation. The estimates are reported as differences between groups' percentages (treatments or time points) and corresponding 95% confidence interval (95% CI). All analyses assumed a significance level of 5%.

**Results** A total of 32 patients were selected. Mean PPD (mm) reduced from 6.23 (6.06–6.40) to 3.33 (3.06–3.61) at T2 for GBT, and from 6.21 (6.04–6.38) to 3.32 (3.11–3.53) at T2 for SRP. Both treatments reached a comparable percentage of closed pockets at T1 (77.9% for GBT vs. 80.1% for SRP, *p* = 0.235) and T2 (84.1% for GBT vs. 84.4% for SRP, *p* = 0.878), with no statistically or clinically significant difference. GBT and traditional SRP with ultrasonic and hand instruments reach satisfactory clinical results in the active treatment of patients with stages III and IV periodontitis, with comparable rates of closed pockets and treatment time.

**Conclusion** GBT is a suitable option in the active phase of periodontitis management in patients with stages III and IV periodontitis.

## Introduction

Scaling and root planing (SRP) with ultrasonic and manual instruments is still the gold standard nonsurgical therapy for periodontal disease. 1 The therapy aims to reach health on the reduced periodontium, with the primary end point being the closure of periodontal pockets. A closed periodontal pocket is defined by pocket probing depth (PPD) ≤ 4 mm and the absence of bleeding on probing (BOP) if PPD = 4 mm. 2 However, SRP is known to have undesired effects on hard and soft tissues, 3 in particular, removal of radicular cementum, 4 roughening of the tooth surface, 5 posttreatment dentinal hypersensitivity, 6 and gingival recession (REC). 7 As the need to remove “infected cementum” has been long disproven, 3 a shift from SRP to the more conservative root surface debridement has been recommended, involving the predominant use of delicate ultrasonic tips instead of manual instrumentation. Ultrasonic debridement can provide the same clinical resolution of disease obtained via traditional root planing 8 9 with further advantages such as less chair time, minimal discomfort, and less local anesthesia. 8 Air polishing can remove stains biofilm, and plaque through a jet of air, water, and low-abrasiveness powders. Full-mouth air polishing with low-abrasiveness powders such as erythritol was also suggested as a minimally invasive 10 11 12 13 14 and clinically effective alternative for biofilm removal in the treatment of gingivitis 15 and during supportive periodontal therapy 10 with the additional advantages of being faster and considered more comfortable by the patients. 10 16 17 18 Air polishing seems to be especially time efficient during maintenance of residual pockets: a short 5-second application of subgingival air polishing can achieve the same clinical result as 1.4 minutes of ultrasonic scaling. 17 19 During regular prophylaxis appointments, it can save up to 9% of appointment time. 15 To date, a few studies have investigated full-mouth air polishing in the initial treatment of patients with periodontitis using a variety of protocols, and the results are still controversial. 20 21 22 23 24 A recently introduced protocol known by the commercial name of guided biofilm therapy (GBT) unifies the techniques above and involves thorough patient assessment, the application of a plaque disclosing solution through which the patient is motivated about home oral hygiene, supra- and subgingival air polishing with erythritol powder including a special tip for pockets, followed by ultrasonic and, if required, manual pocket debridement. GBT resulted as efficient and time-saving in a recent study on supportive periodontal treatment. 25 However, at this stage, to the best of our knowledge, no studies have applied GBT in the initial phase of therapy for periodontal disease.

The aim of the present randomized controlled clinical trial is to compare the clinical results of GBT protocol with traditional SRP in the initial phase therapy of patients with stages III and IV periodontitis. In particular, the percentage of pockets reaching PPD ≤ 4 mm with no BOP after therapy was evaluated. It is hypothesized that both treatments will provide comparable results.

## Materials and Methods

### Study Design and Population

The present study was a single-blinded, randomized, split-mouth, controlled clinical trial, approved by the ethics committee of ASST - Spedali Civili di Brescia (Italy) with protocol number 2519, and conducted following the Declaration of Helsinki. The study was registered in ClinicalTrials.gov (BLINDED).

Patients were selected from the population afferent to the Dental School (BLINDED)

The inclusion criteria were as follows:

- Age between 18 and 75 years old.
- Systemically healthy.
- Diagnosis of stages III and IV periodontitis. 26
- At least six experimental sites per quadrant with PPD >4 mm and <10 mm and loss of clinical attachment level (CAL) ≥ 3 mm.
- At least five teeth per quadrant.

The exclusion criteria were as follows:

- Systemic diseases such as diabetes, heart disease, and cancer.
- Respiratory diseases such as chronic obstructive pulmonary disease and asthma.
- The use of antibiotics within 3 months prior to the start of the study.
- Pregnant or lactating.
- Allergy to chlorhexidine or erythritol.
- Smoking >10 cigarettes per day.
- Unwillingness to undergo the proposed treatment and recalls.

All participants signed a written informed consent before the beginning of the study.

### Sample Size Calculation

The sample size for the primary outcome (proportion of PPD <4 mm BOP negative) was computed through simulation assuming a noninferiority trial with a 2 × 2 crossover design for proportions. Data were simulated (B = 1,000 simulations) assuming three random intercepts (subject, tooth, and site) and a within-subject random slope (the within-subject treatment effect). Variance estimates for the random effects were computed from existing data on a similar setting. Assuming a significance level of 5%, a tolerance margin of 10%, and a sample size of 32 subjects will provide an 80% power. All the calculations were performed using R (R version 4.1.1, R Core Team (2020). R: A language and environment for statistical computing. R Foundation for Statistical Computing, Vienna, Austria. URL https://www.Rproject.org/ ).

### Randomization

Considering potential drop outs after phase I randomization to treatment phase (phase I) and maintenance phase (phase II) was performed in a two-step procedure. Subjects were randomized to an AB scheme with varying sequence of treatment administration to the two split-mouth areas (or maintenance therapy). Randomized patient allocation to either test or control intervention was performed centrally using *adhoc* software (R version 4.1.1, R Core Team (2020). R: A language and environment for statistical computing. R Foundation for Statistical Computing, Vienna, Austria. URL https://www.Rproject.org/ ), using a blocked randomization scheme to achieve balanced treatment groups within center. Sealed opaque envelopes were used, and opened just before the commencement of the treatment.

### Interventions

Patient selection and collection of clinical parameters at baseline, 6 weeks (T1), and 3 months (T2) were performed by the same expert periodontist blinded to the intervention. The following parameters were collected at baseline, T1, and T2: PPD, REC, CAL, BOP, and plaque index (PI). All the parameters were collected in six points per tooth with probe UNC-PCP 15 (Hu-Friedy, Chicago, Illinois, United States). BOP and PI were collected as dichotomous index (presence/absence). 27 Radiographs and clinical photos were also collected at baseline. Experimental sites were selected, defined as sites with PPD >4 and <10 mm without suppuration. Interventions were performed by the same two experienced operators, responsible for treatment delivery only, to ensure blinding. Randomization was assured via sealed opaque envelopes, allocating quadrants I to IV and II to III to either test or control treatment in agreement with the split-mouth design of the study, opened by the operators just before the commencement of the therapy. The treatment was performed over 2 days to limit the chair time and increase patient comfort. Quadrants I to IV were treated on the first day, and quadrants II to III on the second day. Treatment time was measured starting from when the operator picked up the first instrument, and the session ended when the clinician was satisfied with the outcome.

In all subjects, soft tissue protection was obtained with a retractor (OptraGate, Ivoclar Vivadent), and a Teflon membrane was applied on the quadrants not to be treated on the day. The remaining quadrants received the application of a disclosing agent (Mira-2-Ton, Hager & Werken, Duisburg, Germany) to guide and optimize plaque removal. 28

**Fig. 1:**
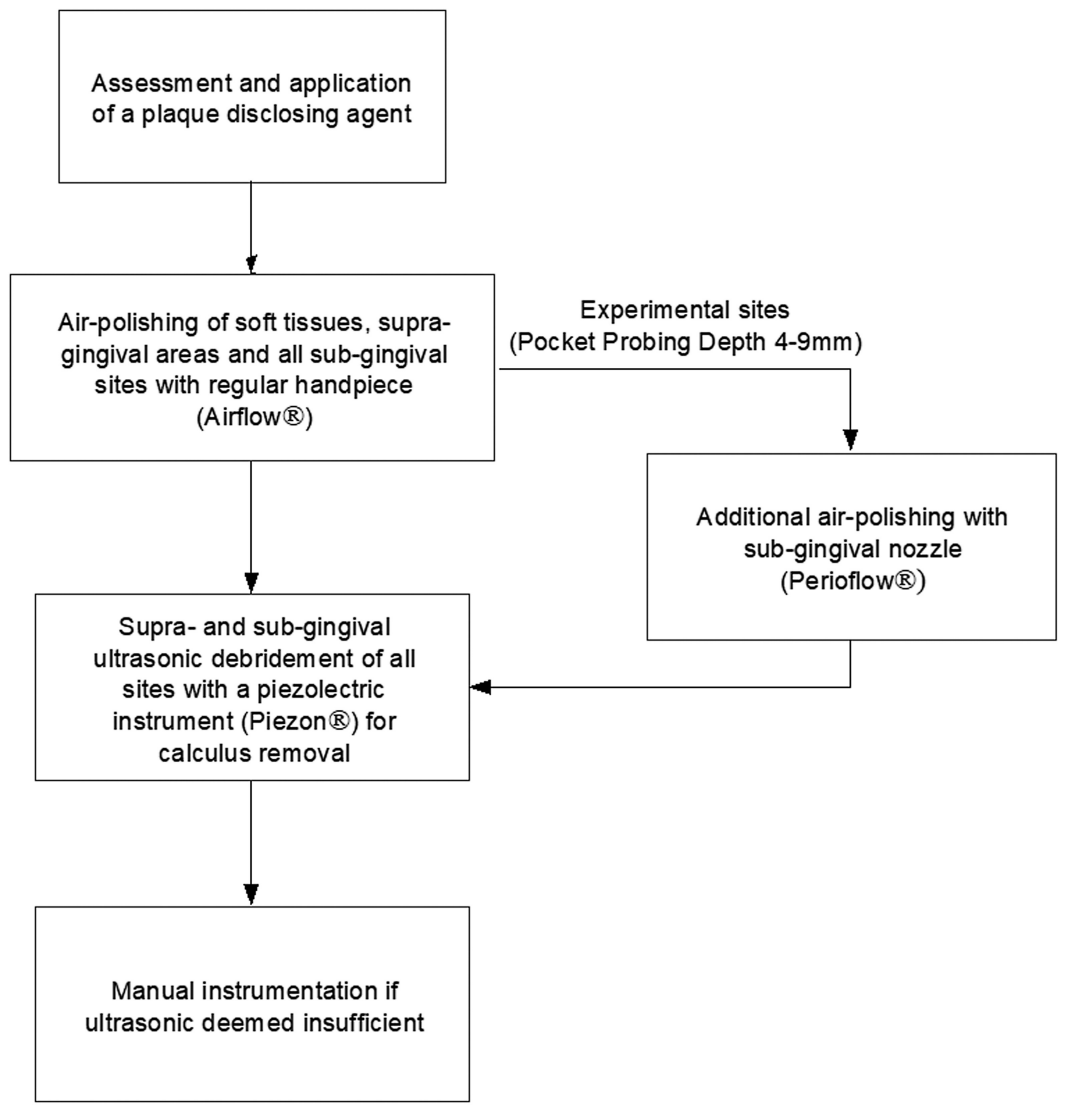
Guided Biofilm Therapy protocol.

The quadrants allocated to the test intervention received the following treatment ( Fig. 1 ):

- Soft tissues (gingival area, tongue, and palate), the supra-gingival areas, and all subgingival sites were air polished (Airflow Prophylaxis Master, EMS, Nyon, Switzerland) with a regular handpiece and erythritol + chlorhexidine powder (PLUS powder, EMS).
- The experimental sites received additional air polishing with a specifically designed subgingival tip (Perioflow, EMS) conveying erythritol + chlorhexidine powder (PLUS powder, EMS).
- Once the air-polishing phase was completed, residual supra- and subgingival calculus were detected visually and through probing, and removed full-quadrant, experimental sites included, by piezoceramic instrumentation with dedicated tip (PerioSlim, Perio, A, PL4 and PL5 tips, Airflow Prophylaxis Master, EMS).
- Experimental sites were checked for any residual calculus with an explorer probe (American Eagle, Montana, United States), and further gentle debridement was provided with either ultrasonic or manual instruments if required. Manual instrumentation was applied if the clinician determined the ultrasonic instrumentation insufficient for calculus removal; however, no root planing was performed.

This procedure is known with the name of GBT. 23 25

The quadrants allocated to control received SRP as follows:

- Full-quadrant supra- and subgingival areas, including experimental sites, received piezoceramic instrumentation with dedicated tips (PerioSlim, Perio, A, PL4 and PL5 tips, Airflow Prophylaxis Master, EMS).
- Experimental sites also received manual scaling with minicurettes 7–8, 11–12, 13–14 (American Eagle, Montana).
- Final plaque and stain removal were performed with a rubber cup (ProCup Soft Light Blue, Kerr, Bioggio, Switzerland) and abrasive paste (Cleanic, Kerr) with a relative dentin abrasion value of 27.

**Fig. 2:**
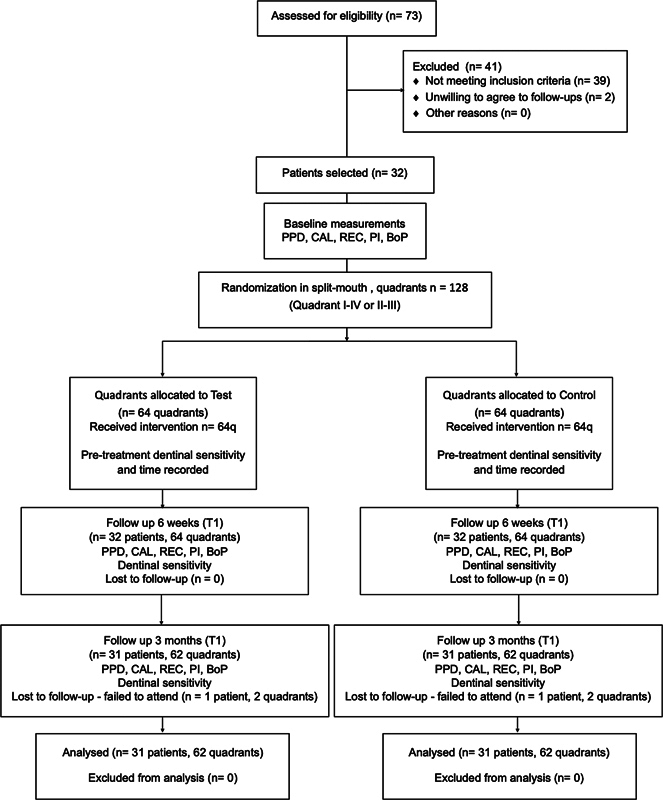
CONSORT study flowchart. BOP, bleeding on probing; CAL, clinical attachment loss; PI, plaque index; PPD, pocket probing depth; REC, recession.

At the end of the session, the patients of both groups received oral hygiene instructions on manual toothbrushing with a soft toothbrush and the use of interdental brushes, which were provided and demonstrated. The patients were instructed to clean all quadrants in the same manner. The study protocol is displayed in Fig. 2 .

### Outcomes

The primary outcome was the number of experimental sites (PPD >4 and <10 mm) becoming closed pockets (PPD ≤ 4 mm and BOP negative) at 6 weeks (T1) and 3 months (T2) after initial therapy. Secondary outcomes were PPD, REC, CAL, BOP, and PI variations at the experimental sites and treatment time. A 10% difference in the primary outcome between the two protocols was set as the threshold to define the inferiority/noninferiority of the test treatment.

### Statistical Analysis

Quantitative variables were summarized using mean (standard deviation SD), while qualitative variables were reported as counts (percentages). Baseline summary statistics were computed on all sites. All the models were fitted on sites classified as “experimental” at baseline (PPD >4 and <10 mm, no suppuration). The primary outcome, the number of closed pockets (PPD ≤ 4 mm BOP negative), was modeled using a generalized estimating equation (GEE) with binomial distribution and identity link. The estimates are reported as differences between groups' percentages (treatments or time points) and corresponding 95% confidence interval (95% CI). Both PPD and CAL were modeled using GEE with Gamma distribution and identity link. This model allows us to have a better fit for the distribution of residuals, highly non-Gaussian, and to have estimates reported as differences. Due to the high number of zeros present in REC measurement, we modeled it using a gamma generalized linear mixed model accounting for zero inflation (hurdle model). All analyses assumed a significance level of 5% and were performed using statistical R (R version 4.1.1, R Core Team (2020). R: A language and environment for statistical computing. R Foundation for Statistical Computing, Vienna, Austria. URL https://www.Rproject.org/ ).

## Results

A total of 73 patients were screened for the study, and 32 patients were selected. During the study, one patient was excluded (dropout) due to failure to attend the T2 appointment ( Fig. 2 ).

Demographic characteristics and baseline periodontal parameters of the study population are presented in Table 1 . Thirteen females and Nineteen males completed the study. The mean age was 46.34 ± 11.67 SD. Twelve participants were smokers. Baseline overall periodontal parameters of the population at a site level are reported and divided into GBT and SRP groups ( Table 2 ). A separate analysis of the baseline periodontal parameters at experimental sites only, defined as sites with PPD >4 and <10 mm without suppuration, is shown in Table 3 . GBT and SRP groups were comparable for all considered parameters.

**Table 1:** Study population demographics, baseline overall sites periodontal parameters, and periodontal parameters for GBT and SRP groups for all sites

			GBT ( *N* = 2,430)	SRP ( *N* = 2,484)	*p* -Value
Gender	Male	19 (59.4%)			
	Female	13 (40.6%)			
Age	Mean (SD)	46.34 (11.67)			
	Median (MAD)	46.50 (6.50)			
	Range	22.00–70.00			
Smoking	Yes	12 (37.5%)			
	No	20 (62.5%)			
PI (%)	Mean (SD)	75.01 (36.05)	74.1%	73.4%	0.54
	Median (MAD)	100.00 (0.00)			
	Range	0.00–100.00			
BOP (%)	Mean (SD)	79.78 (26.56)	79.3%	79.9%	0.63
	Median (MAD)	94.99 (5.01)			
	Range	14.49–100.000			
REC (mm)	Mean (SD)	0.34 (0.87)	0.55 (1.26)	0.51 (1.22)	0.87
	Median (MAD)	0.00 (0.00)			
	Range	0.00–3.00			
PPD (mm)	Mean (SD)	5.03 (2.07)	4.38 (2.33)	4.41 (2.23)	0.16
	Median (MAD)	5.00 (1.50)			
	Range	2.00–9.00			
	≤4		1,370 (56.4%)	1,356 (54.6%)	0.23
	>4		1,060 (43.6%)	1,128 (45.4%)	

Abbreviations: BOP, bleeding on probing; GBT, guided biofilm therapy; MAD, median absolute deviation; *N* , number of sites analyzed; PI, plaque index; PPD, pocket probing depth; REC, recession; SD, standard deviation; SRP, scaling and root planing.

**Table 2:** Baseline periodontal parameters of all examined sites for GBT and SRP groups

		GBT ( *N* = 2,430)	SRP ( *N* = 2,484)	*p* -Value
PPD (mm) mean (SD)		4.38 (2.33)	4.41 (2.23)	0.16
PPD (mm)	≤4	1,370 (56.4%)	1,356 (54.6%)	0.23
	>4	1,060 (43.6%)	1,128 (45.4%)	
REC (mm) mean (SD)		0.55 (1.26)	0.51 (1.22)	0.87
BOP	Yes	1,927 (79.3%)	1,985 (79.9%)	0.63
	No	503 (20.7%)	499 (20.1%)	
CAL (mm) mean (SD)		4.94 (2.64)	4.93 (2.46)	0.25
PI	Yes	1,800 (74.1%)	1,823 (73.4%)	0.54
	No	630 (25.9%)	661 (26.6%)	

Abbreviations: GBT, guided biofilm therapy; MAD, median absolute deviation; N, number of sites analyzed; SD, standard deviation; SRP, scaling and root planing.

**Table 3:** Mean values for PPD, CAL, REC, BOP, and PI at baseline, T1, and T2 for experimental sites only, and the ratio between different time points and test control

		Baseline	T1	T2	Ratio T1–T0	*p* -Value	Ratio T2–T0	*p* -Value
PPD (mm) (95% CI)	GBT	6.23 (6.06, 6.40)	3.55 (3.28, 3.81)	3.33 (3.06, 3.61)	−2.68 (−3.08, −2.28)	<0.01	−2.894 (−3.299, −2.489)	<0.01
	SRP	6.21 (6.04, 6.38)	3.45 (3.26, 3.64)	3.32 (3.11, 3.53)	−2.76 (−3.078, −2.44)	<0.01	2.892 (–3.233, 2.551)	<0.01
	Ratio GBT–SRP	0.016 (−0.118, 0.151)	0.095 (−0.041, 0.232)	0.014 (−0.103, 0.131)				
	*p* -Value	0.812	0.171	0.812				
CAL (mm) (95% CI)	GBT	6.74 (6.43, 7.05)	4.37 (3.88, 4.86)	4.09 (3.58, 4.61)	–2.37 (–3.08, –1.66)	<0.01	−2.647 (−3.222, −2.071)	<0.01
	SRP	6.68 (6.42, 6.95)	4.27 (3.85, 4.68)	4.10 (3.67, 4.52)	–2.42 (−3.03, −1.81)	<0.01	−2.588 (−3.123, −2.054)	<0.01
	Ratio GBT–SRP	0.057 (−0.134, 0.248)	0.108 (−0.103, 0.318)	−0.002 (−0.189, 0.185)				
	*p* -Value	0.560	0.317	0.987				
REC (mm) (95% CI)	GBT	2.32 (2.04, 2.59)	2.30 (2.05, 2.55)	2.25 (1.99, 2.50)	−0.012 (−0.281, 0.257)	0.998	−0.068 (−0.340, 0.204)	0.889
	SRP	2.32 (2.03, 2.62)	2.15 (1.89, 2.42)	2.20 (1.93, 2.47)	−0.173 (−0.433, 0.087)	0.298	−0.127 (−0.392, 0.138)	0.572
	Ratio GBT–SRP	-0.008 (-0.264, 0.249)	0.153 (−0.031, 0.336)	0.051 (−0.144, 0.246)				
	*p* -Value	0.953	0.102	0.608				
BOP (95% CI)	GBT	91.12 (85.56, 96.68)	38.95 (30.79, 47.11)	31.06 (25.03, 37.08)	−52.17 (−64.62, −39.72)	<0.01	−60.06 (−70.12, −50.00)	<0.01
	SRP	91.60 (86.23, 96.98)	36.19 (28.18, 44.21)	32.58 (26.33, 38.83)	−55.41 (−67.24, −43.58)	<0.01	−59.03 (−69.25, −48.80)	<0.01
	Ratio GBT-SRP	−0.48 (−3.52, 2.55)	2.76 (−3.95, 9.46)	−1.52 (−6.85, 3.80)				
	*p* -Value	0.754	0.420	0.575				
PI (95% CI)	GBT	83.55 (73.06, 94.05)	33.70 (22.91, 44.49)	31.48 (24.16, 38.81)	−49.85 (−68.60, −31.09)	<0.01	−52.07 (−67.73, −36.40)	<0.01
	SRP	82.65 (72.80, 92.50)	33.58 (22.01, 45.15)	29.23 (20.74, 37.72)	−49.07 (−68.09, −30.05)	<0.01	−53.42 (−69.25, −37.59)	<0.01
	Ratio GBT–SRP	0.90 (−3.36, 5.17)	0.12 (−4.48, 4.72)	2.25 (−3.41, 7.91)				
	*p* -Value	0.678	0.959	0.436				

Abbreviations: BOP, bleeding on probing; CAL, clinical attachment loss; CI, confidence interval; GBT, guided biofilm therapy; PI, plaque index; PPD, pocket probing depth; REC, recession; SD, standard deviation; SRP, scaling and root planing.

Note: bold values shows the *p* values reaching statistical significance.

Table 4 shows the percentage of experimental sites reaching PPD ≤ 4 mm and no BOP at 6 weeks and 3 months after the treatment. Both GBT and SRP obtained a comparable percentage of closed pockets at T1 (77.9% [70.9; 84.9] vs. 80.1% [75.3; 85.00], *p* = 0.235) and T2 (84.1% [77.7; 90.5] vs. 84.4% [79.0; 89.7], *p* = 0.878), with no statistically or clinically significant difference.

**Table 4:** Percentage of test sites (PPD >4 and <10 mm) becoming closed pockets (PPD ≤4mm BOP negative) at 6 weeks (T1) and 3 months (T2) after initial therapy

Closed pockets (%)				
	GBT	SRP	Delta GBT–SRP	*p* -Value
T1 (95% CI)	77.9 (70.9, 84.9)	80.1 (75.3, 85.00)	−2.23 (−5.91, 1.45)	0.235
T2 (95% CI)	84.1 (77.7, 90.5)	84.4 (79.0, 89.7)	−0.25 (−3.46, 2.96)	0.878
Delta T1–T2 (95% CI)	6.20 (−3.41, 15.80)	4.22 (−3.13, 11.57)		
*p* -Value	0.206	0.206		

Abbreviations: CI, confidence interval; GBT, guided biofilm therapy; SRP, Scaling and Root Planing.

Note: Delta T1–T2 represents the intragroup variation in the percentage of closed pockets between the two time points, whilst delta GBT–SRP the intergroup variation at the two time points.

Table 3 shows the mean values for PPD, CAL, REC, BOP, and PI at T0, T1, and T2 for experimental sites only. Both GBT and SRP treatment resulted in a significant improvement of the periodontal parameters. Mean PPD (mm) reduced from 6.23 (6.06–6.40) to 3.55 (3.28–3.81) at T1 and 3.33 (3.06–3.61) at T2 for GBT, and from 6.21 (6.04–6.38) to 3.45 (3.26–3.64) at T1 and 3.32 (3.11–3.53) at T2 for SRP. Again, the difference between treatments was not significant. The treatments also resulted in comparable changes in CAL, REC, BOP, and PI. While there was a significant improvement between T0, T1, and T2 for PPD, CAL, BOP, and PI, the REC did not show a significant change at any of the time points. This is confirmed by the overall analysis of the study population in Table 5 , where the ratio between baseline and T1 and baseline and T2 reveals a statistically significant improvement of all parameters except for REC.

**Table 5:** Overall full-mouth periodontal parameters (mean values and standard deviation), and ratio between different time points

	Baseline	T1	T2	T1/baseline	*p* -Value	T2/baseline	*p* -Value
PPD (mm) (95% CI)	4.40 (4.11, 4.68)	2.74 (2.55, 2.94)	2.65 (2.46, 2.85)	−1.653 (−2.041, −1.264)	**<0.01**	−1.742 (−2.125, −1.359)	**<0.01**
CAL (mm) (95% CI)	4.93 (4.56, 5.30)	3.49 (3.11, 3.88)	3.37 (2.97, 3.76)	−1.438 (−2.034, −0.841)	**<0.01**	−1.565 (−2.068, −1.061)	**<0.01**
REC (mm) (95% CI)	2.35 (2.08, 2.62)	2.28 (2.02, 2.55)	2.33 (2.07, 2.60)	−0.071 (−0.181, 0.039)	0.266	−0.019 (−0.132, 0.094)	0.890
BOP (95% CI)	0.80 (0.70, 0.89)	0.28 (0.22, 0.34)	0.24 (0.19, 0.29)	−0.517 (−0.641, −0.393	**<0.01**	−0.559 (−0.673, −0.444)	**<0.01**
PI (95% CI)	0.74 (0.61, 0.86)	0.29 (0.19, 0.38)	0.24 (0.18, 0.31)	−0.451 (−0.627, −0.275)	**<0.01**	−0.494 (−0.650, −0.339)	**<0.01**

Abbreviations: BOP, bleeding on probing; CAL, clinical attachment loss; CI, confidence interval; PI, plaque index; PPD, pocket probing depth; REC, recession.

The average time needed to complete the GBT and SRP treatments is shown in Table 6 . The times are comparable, with an average of 4.31 minutes of difference between the two protocols.

**Table 6:** Average time needed to complete the test and control treatments

	Time	*p* -Value
GBT (95% CI)	55.44 (48.90, 61.97)	–
SRP (95% CI)	51.13 (44.71, 57.54)	–
GBT–CTR ratio (95% CI)	4.31 (–0.25, 8.88)	0.0637

Abbreviations: CI, confidence interval; GBT, guided biofilm therapy; SRP, scaling and root planing.

## Discussion

This split-mouth, randomized, controlled study evaluated the clinical outcome of subgingival air polishing with erythritol powder and ultrasonic calculus removal during the initial treatment of patients with stages III and IV periodontitis. Traditional SRP served as a control treatment for the study.

Both GBT and SRP treatment significantly improved the periodontal parameters, both from the statistical and clinical points of view. At 3 months after initial therapy, 84.1% of the GBT experimental sites and 84.4% of the SRP experimental sites reached PPD ≤4 mm with no BOP if the PPD was 4 mm, falling under the definition of closed pockets. 2 Given that less than 10% difference is noted in the primary outcome between the two protocols, the GBT protocol was demonstrated as noninferior to the SRP gold standard nonsurgical therapy.

The results are very positive if we consider the existing literature about the efficacy of periodontal nonsurgical therapy. Wennström et al (2005) 8 compared the efficacy of traditional quadrant SRP and a new protocol involving full-mouth ultrasonic debridement in patients with periodontitis. At the 3-month re-examination, SRP achieved a pocket closure of 66%, while ultrasonic debridement achieved 58% pocket closure. The percentage increased to 77 and 74%, respectively, at 6 months. In the present study, a more significant decrease in PPD (1.8 mm reduction vs. 2.68 mm for GBT and 2.76 mm for SRP in the present study) and a higher percentage of pocket closure (more than 80% in the present study) was obtained in a shorter period for both protocols considered. One would think the difference could be due to the fact that, in the present study, we did not consider experimental sites pockets with PPD > 10 mm. At the same time, there was no upper PPD limit in Wennström et al (2005), 8 as it is known that deeper pockets are more challenging to debride effectively, and the extent of residual calculus is directly related to pocket depth. 29 The pocket closure in the present study also resulted higher than the weighted mean pocket closure estimated by a systematic review by Suvan et al (2020) 30 on the subgingival instrumentation (manual/sonic/ultrasonic) for the treatment of periodontitis, calculated at 74% at 6/8 months after the treatment. Therefore, the results showed that the participants in this study seem to have reached a more satisfactory result than average in a much shorter time. A possible explanation may lie in the heterogeneity of the studies included in the review, with various instruments, design and technology, protocols, and combinations of instruments, which might have produced a range of clinical outcomes. Also, the parameter “pocket closure” was not consistently reported in the studies considered.

All other periodontal parameters (CAL, BOP, and PI) improved significantly, with no difference between the groups, except for REC, which did not show any significant change during the study. The authors hypothesize the reason being intentional removal of cementum was performed, even when manual instrumentation was applied, but just gentle debridement of the root surface, which possibly preserves the precious cementum regenerative potential encourages gain of attachment rather than REC. 31 32 Interestingly, while the mean PPD value in experimental sites is very similar for both groups at both time points, the percentage of closed pockets results slightly higher (even if not significant) in the SRP group at T1 (80.1% for SRP vs. 77.9% for GBT), then it levels at T2 (84.4% for SRP vs. 84.1% for GBT).

BOP at experimental sites was at T2 31.06% for GBT and 32.58% for SRP, revealing the presence of sites that might need further treatment. According to the new periodontal classification of 2018 2 : “Periodontal stability is characterized by successful treatment through control of local and systemic risk factors, resulting in minimal (<10% of sites) BOP, no probing depths of 4 mm or more that bleed on probing, optimal improvement in other clinical parameters, and lack of progressive periodontal destruction.” In the present study, the overall BOP at T1 and T2 seems far from the BOP <10% goal ( Table 3 ). However, a slight improvement is noticeable between T1 and T2. Therefore, stronger motivation and oral hygiene instruction, together with supportive periodontal therapy, could further reduce BOP. Once again, when analyzing the experimental sites only, the results at T2 seem better than the ones obtained by Wennström et al (2005), 8 where they reduced the BOP at experimental sites to 44 and 48% with SRP and ultrasonic debridement, respectively. Suvan et al (2020) 30 estimated a BOP reduction of 62% at 6/8 months, very close to the 60.06 and 59.03% reduction obtained in the GBT and SRP groups, respectively, at only 3 months posttreatment.

To date, five other studies have investigated the application of air polishing in the active treatment of periodontal patients, 20 21 22 23 24 four of which reached a similar conclusion that no clinical advantage could be observed, and one found a greater reduction in the number of pockets with PPD ≥5 mm. However, a comparison is difficult due to the great variability of materials and protocols applied. In Park et al (2018) 21 and Jentsch et al (2020), 24 the same erythritol + chlorhexidine powder as the present study was applied. However, in both studies, air polishing was applied after the initial manual and sonic instrumentation, against the manufacturer's recommendations, and was limited to the test sites, as opposed to a full-mouth application, including supragingival areas and healthy sulci. Tsang et al (2018) 22 and Caygur et al (2017) 20 utilized a glycine-based powder, again after ultrasonic and manual instrumentation, and in limited areas. In summary, these studies applied air polishing as a mere adjunctive therapy to traditional subgingival instrumentation.

What differentiates the present study from all the ones above is the more prominent use of air polishing in the GBT protocol, compared with the traditional SRP. The authors decided to apply the full-mouth air-polishing concept as conceived by Flemmig et al (2012) 12 to the GBT group, not limiting air polishing to the treatment of pockets only. This is similar to the protocol applied in a previous study 23 investigating the additional use of subgingival air polishing with erythritol powder in the treatment of periodontitis patients. However, in Mensi et al (2021), 23 the control group also received supra- and juxta-gingival air polishing without the additional subgingival air polishing at the experimental sites. This time, the authors wanted to stick to the traditional SRP protocol as a control treatment.

Flemmig et al (2012) 12 achieved a significant reduction of subgingival biofilm with their full-mouth air-polishing protocol with glycine powder and a positive microbiota shift. However, their control protocol only involved manual SRP, with no sonic/ultrasonic instrumentation application. It is possible that, in the present study, the application of piezoceramic instrumentation in both GBT and SRP groups led to a better removal of subgingival plaque than what one can obtain with manual instrumentation only. Ultrasonic instrumentation seems to allow better access to deep pockets and furcation areas, 33 34 and the water flow can remove part of the bacterial residuals and endotoxins. 35

Finally, this study failed to reveal any statistically or clinically significant difference in treatment time between GBT and SRP. The average time difference between GBT and SRP was 4.31 minutes in favor of GBT. However, the CI reveals evident variability. This result slightly differs from other studies applying GBT. Mensi et al (2022) 15 found a reduction of treatment time of 9.5% in patients with gingivitis, and Park et al (2021) 36 similarly found a reduction in treatment time when air polishing was applied instead of rubber cup and prophylaxis paste, especially the time required for ultrasonic and manual scaling. Vouros et al (2022) 25 save an average of 5.1 minutes during supportive periodontal therapy using air polishing, compared with traditional protocols. The difference might be due to the patient cohorts in these studies: initial periodontal treatment is usually longer and more demanding than supportive periodontal therapy or general prophylaxis, and calculus removal represents a time-consuming task.

One aspect to not underestimate is the comfort for the treating practitioner during such long debridement sessions: Vouros et al 25 found that GBT caused less hand fatigue and less noise than SRP.

No side effects of air polishing were observed (e.g., swelling, subcutaneous emphysema). This is likely due to the low abrasiveness of the powder used and the strict adherence to manufacturer recommendations, advising against the use of the subgingival nozzle in pockets showing purulent exudate and pockets that have already been treated with a manual/ultrasonic instrument, as this would increase the likelihood of conveying air in the deeper connective tissue. A limitation of the present study was that the operator was very conservative even in the application of the traditional root planing with manual instruments, possibly influencing the results obtained in the control group.

Another limitation may lie in the selection of a split-mouth design. While this design eliminates the interindividual variability of the clinical results, the risk of carry-over effect is higher, 37 especially in a noninferiority trial such as the present one. It is also possible that the chlorhexidine contained in the powder as a preservative, while a low amount (0.3%) influenced the healing in the control quadrants with its delayed release from the mucosae. Future parallel-design clinical trials with bigger sample sizes which will allow subanalysis for other variables, such as smoking status, might help clarify the results above.

Finally, from the point of view of daily practice, clinicians should keep in mind that the choice to adopt new technology and protocols requires thorough consideration, especially in terms of the learning curve, which might initially impact the treatment effectiveness and outcomes, and the costs involved in the additional equipment and related education. Running expenses also need to be quantified. While a general cost analysis is complicated and needs to be performed on an individual base, it appears that ongoing treatment with air polishing might not weigh too heavily on the clinic's finances. 38

## Conclusion

GBT allows obtaining the same clinical results as SRP in the initial treatment of patients with stages III and IV periodontitis, in terms of percentage of pocket closure and improvement in the periodontal parameters. The treatment time was comparable. The choice of one or the other approach will be made according to expertise of the operator with technology and his/her comfort.
